# Fall predictors beyond fall risk assessment tool items for acute hospitalized older adults: a matched case–control study

**DOI:** 10.1038/s41598-021-81034-9

**Published:** 2021-01-15

**Authors:** Hye-Mi Noh, Hong Ji Song, Yong Soon Park, Junhee Han, Yong Kyun Roh

**Affiliations:** 1 Department of Family Medicine, College of Medicine, Hallym University Sacred Heart Hospital, Hallym University, Anyang, 14068 Republic of Korea; 2 Department of Family Medicine, Chuncheon Sacred Heart Hospital, Hallym University College of Medicine, Chuncheon, 24253 Republic of Korea; 3 Department of Statistics and Institute of Statistics, Hallym University, Chuncheon, 24252 Republic of Korea; 4 Department of Family Medicine, Kangnam Sacred Heart Hospital, 1, Singil-ro, Yeongdeungpo-gu, Seoul, 07441 Republic of Korea

**Keywords:** Epidemiology, Geriatrics, Predictive markers

## Abstract

We investigated whether clinical factors including comorbidities, medications, and laboratory results predict inpatient fall risk in older adults. The participants in this case–control study included hospitalized older adults with acute conditions who had falls during their hospital stay (case group) and 410 hospitalized older adults who did not experience falls (control group). Data on medical history, fall risk assessment (Morse Fall Scale; MFS), medications, and laboratory results were obtained. Conditional logistic regression analysis was performed to estimate the association between clinical factors and falls. Receiver operating characteristic curves and area under the curve (AUC) were used to determine whether clinical factors could discriminate between fallers and controls. We evaluated three models: (M1) MFS, (M2) M1 plus age, sex, ward, and polypharmacy, and (M3) M2 plus clinical factors. Patients with diabetes mellitus or MFS scores ≥ 45 had the highest risk of falls. Calcium channel blockers, diuretics, anticonvulsants, and benzodiazepines were associated with high fall risk. The AUC of the three models was 0.615, 0.646, and 0.725, respectively (M1 vs. M2, *P* = 0.042 and M2 vs. M3, *P* < .001). Examining clinical factors led to significant improvements in fall prediction beyond that of the MFS in hospitalized older adults.

## Introduction

Inpatient falls are serious adverse events that increase the length of hospital stays, medical costs, and mortality^1^. It has been reported that advanced age is a risk factor for falls, with higher rates of falls and injurious falls among older hospitalized patients than their younger counterparts^2^. To prevent older patients from accidentally falling while hospitalized, it is necessary to assess the risk of falls and accordingly provide the necessary interventions. Several fall risk assessment tools such as the Morse Fall Scale (MFS)^3^, St Thomas’s Risk Assessment Tool in Falling Elderly Inpatients^4^, and the Hendrich Fall Risk Model have been utilized in hospitals^5^. Of these, the MFS is the most popular because of its ease of use; nurses can perform the rating in under three minutes. The MFS consists of six items, each of which is scored between either 0–15 points or 0–30 points: history of falling within three months, secondary diagnosis, intravenous (IV) therapy/heparin lock, use of ambulatory aid, gait, and mental status. Patients with total MFS scores ≥ 45 are defined as high-risk^6^. However, the models’ prediction abilities vary when applied in hospitalized patients with acute conditions^7^, and they lack consideration of various clinical factors. In acute care hospitals, patients’ medications and medical conditions can change rapidly according to treatment. This indicates that adding factors such as comorbidities, fall risk-increasing drugs (FRID), and laboratory results to previous tools may improve their ability to predict inpatient falls. Recently, the Agency for Healthcare Research and Quality (AHRQ) suggested that evaluating medications, disease states, laboratory results, and patients’ education levels can prevent falls in hospitals^8^.

Furthermore, studies have reported that certain medications increase the risk of inpatient falls in hospitalized patients with acute conditions. A matched case–control study at a tertiary care center in the US reported that psychotropic agents such as benzodiazepines, antiepileptics, antidepressants, antipsychotics, hypnotics, central nervous system stimulants, narcotics, and antiparkinsonian agents were associated with the risk of injurious falls in the inpatient setting^9^. A retrospective cohort study in two US hospitals demonstrated that a higher number of FRID, higher comorbidity predisposition including 11 past diagnoses related to falls (confusion, disorientation, and impulsivity; dizziness and vertigo; hallucinations; visual impairment; hearing loss; vestibular dysfunction; language impairment; orthostatic hypotension; cerebrovascular accident; Parkinson’s disease; and seizure disorder), and a history of falling increased the risk of inpatient falls^10^.

In general, there has been little research focusing on older hospitalized patients, although one German study reported that benzodiazepines, serotonin-noradrenalin reuptake inhibitors, and Z-drugs (zopiclone, eszopiclone, zaleplon, and zolpidem) were associated with inpatient falls among older adults (aged ≥ 65 years). Additionally, they demonstrated that the presence of hyponatremia and leukocytosis on admission increased the risk of falls^11^. Other studies have reported that abnormal laboratory values such as those indicating anemia or hyponatremia are associated with inpatient falls^12,13^.

Considering these recent findings, we investigated whether clinical factors including comorbidities, FRID, and laboratory results before falls were associated with inpatient falls. We extracted comprehensive clinical data from a clinical data warehouse (CDW) of electronic health records and evaluated the risk-discriminative performance between MFS alone and MFS plus clinical factors. We used a case–control study design that matched age, admission date, ward, and length of stay before falls among older hospitalized patients in a Korean tertiary hospital.

## Methods

### Study population

The study took place at an academic tertiary hospital in a city in Korea. The hospital has 13 ward units and 710 inpatient acute care beds. The facility provides medical services in 33 departments including internal medicine, family medicine, general surgery, neurology, orthopedics, urology, otorhinolaryngology, ophthalmology, pediatrics, psychiatrics, and obstetrics and gynecology. All study participants provided informed consent, and the institutional review board of Hallym University Sacred Heart Hospital (approval number 2018–11-002–001) reviewed and approved the research protocol. All procedures were performed in accordance with the Declaration of Helsinki.

### Fallers and control group

This study used a retrospective case–control design. Falls were identified by the adverse event reports which filled out by the nurses of the department on electronic database. From January 2016 through December 2018, 369 inpatient falls occurred in the study hospital. During the same period, 783,758 beds were occupied, and the fall rate was 4.71 falls per 1,000 occupied bed days. For this study, we included 269 inpatient falls among older patients. If a patient had multiple falls during their hospital stay, we only included data for the first fall. In this manner, 251 fallers were identified. We excluded patients admitted to psychiatric wards (n = 23) and those who did not match the control characteristics (n = 18), resulting in 210 fallers being eligible for this study. The CDW of electronic health records identified one or two controls for each case from those patients who did not experience falls during their hospital stay. Our institution has been using smart CDW since 2016 to analyze big data based on the QlikView Elite Solution (Qlik, Radnor, Pennsylvania, USA)^14^. Matching characteristics identified were age (within two years), admission date (within two weeks), ward, and length of stay before falls. Finally, 210 fallers (57–95 years) and 410 controls (55–97 years) were included in the study.

### Data collection

From the CDW, we extracted data on patients’ demographics, medical histories, fall risk assessments, medications, and laboratory results. At admission, nurses interviewed the patients to obtain their demographic data including smoking status, alcohol consumption, and presence of comorbidities (hypertension, diabetes mellitus, dyslipidemia, heart disease, cerebrovascular disease, malignancy, chronic lung disease, chronic kidney disease, chronic liver disease, osteoporosis, cognitive impairment, Parkinson’s disease, psychiatric disease, urinary disorder, benign prostate hyperplasia, and visual or hearing impairment). Body weight (kg) and height (cm) were measured to the nearest 0.1 kg and 0.1 cm. Body mass index (BMI) was calculated as weight divided by height squared (kg/m^2^).

We compared medications including FRID that were used one and two days before falls in fallers with those in the control group on matched hospital days. FRID was defined based on the AHRQ Fall Prevention Toolkit^8^ and the American Geriatric Society Beers criteria^15^. Beta blockers, calcium channel blockers, angiotensin II receptor antagonists, angiotensin-converting enzyme inhibitors, digoxin, loop diuretics, other diuretics (thiazides or spironolactone), alpha blockers, anticholinergics, antihistamines, weak opioids, opioids, antipsychotics, anticonvulsants, benzodiazepine, hypnotics, anti-dementia drugs, antiparkinsonians, selective serotonin reuptake inhibitors or serotonin-norepinephrine reuptake inhibitors, tricyclic antidepressants, other antidepressants, vasodilating agents, and muscle relaxants were included in FRIDs.

In the study hospital, nurses used the MFS to perform fall-risk assessments at least every two days or whenever a patient’s medical condition changed based on electronic health records. The MFS consists of six items: three with possible answers of ‘yes” or “no” (history of falling within three months, secondary diagnosis, IV therapy/heparin lock); ambulatory aid use with possible answers of “none,” “bed rest,” “nurse assist/crutches,” “cane,” or “walker/furniture”; gait impairment with possible answers of “normal,” “bed rest,” “immobile/weak gait/impaired gait,” and mental status, with possible answers of “oriented to own ability” or “forgets limitations.” Items scores range between 0–15 points and 0–30 points and total MFS scores range from 0 to 125 points. The high-risk group was defined as patients with total MFS scores ≥ 45^16^. Laboratory tests included complete blood count, blood urea nitrogen, creatinine, serum electrolytes, glucose, hemoglobin A1c, aspartate aminotransferase (AST), alanine aminotransferase (ALT), protein, and albumin. Abnormal laboratory values were defined as follows: leukocytosis (white blood cell count > 10,000/uL), anemia (hemoglobin < 13 g/dL in men or < 12 g/dL in women), hypoalbuminemia (albumin < 3.8 g/dL), decreased estimated glomerular filtration rate (< 60 mL/(min*1.73 m^2^)), hyponatremia (sodium < 135 mmol/L), hypokalemia (potassium < 3.6 mmol/L), abnormal liver function test (≥ 2* upper normal limit of AST or ALT), and uncontrolled diabetes mellitus (hemoglobin A1c ≥ 8.0%). We compared MFS score and laboratory values that were obtained immediately prior to falls in the case group with those in the control group on matched hospital days.

### Statistical analysis

Mean (standard deviation) or frequency (percentage) was used to describe patients’ general characteristics. The Student’s *t*-test or Mann–Whitney U test was used to compare continuous variables, and either the chi-square test or Fisher’s exact test was used to compare categorical variables. We estimated odds ratios (OR) and 95% confidence intervals (CIs) to investigate the association between clinical factors and inpatient falls using conditional logistic regression analysis. All variables that were significantly different between fallers and controls, such as BMI, diabetes mellitus, cognitive impairment, MFS score, leukocytosis, hypoalbuminemia, hyponatremia, and polypharmacy (concomitant use of five or more drugs), use of calcium channel blockers, diuretics (thiazides or spironolactone), antipsychotics, anticonvulsants, benzodiazepines, and antiparkinsonians, were included in the model. We evaluated matching covariate-adjusted receiver operating characteristic (ROC) curves and area under the curve (AUC) to determine whether clinical factors could discriminate between fallers and controls^17^. We evaluated three models: (M1) MFS alone, (M2) MFS plus matching covariates (age and ward), sex, and polypharmacy, (M3) M2 plus laboratory values, FRID, and comorbidities. Non-parametric methods were applied according to DeLong et al.^18^ to evaluate differences in AUC estimates. All statistical tests were two-sided, and the significance level was set at *P* < 0.05. All analyses were performed using IBM SPSS Statistics for Windows version 21.0 (IBM Corp., Armonk, NY, USA) and R Studio version 3.6.1.

## Results

### General characteristics of fallers and controls

The age range of the sample was 55–97 years, and the mean age and median age was 73.7 years and 73.0 years, respectively. Comparisons of the general characteristics of fallers and controls included age, sex, height, body weight, BMI, and whether they smoked or drank alcohol. These and the list of comorbidities are shown in Table 1. There were no significant differences in age, sex, number of comorbidities, and visual or hearing impairment between the two groups. Fallers had lower BMI compared to controls (22.8 ± 3.87 and 23.5 ± 3.69, respectively, *P* = 0.009). The prevalence of diabetes mellitus and cognitive impairment was higher among fallers than controls (37.6% and 27.3%, *P* = 0.009; 6.7% and 2.7%, *P* = 0.017; respectively). In addition, fallers had longer hospital days and lower rates of home discharge compared to controls (all *P* < 0.001).

**Table 1:** General characteristics of fallers and matched controls.

	Fallers (n = 210)	Controls (n = 410)	*P* value
Age, years	73.7 ± 8.41	73.6 ± 8.41	0.965
Male, n (%)	125 (59.5%)	240 (58.5%)	0.813
Height, cm	159.9 ± 9.82	160.3 ± 9.16	0.793
Body weight, kg	58.4 ± 11.66	60.5 ± 11.50	0.009
Body mass index, kg/m^2^	22.8 ± 3.87	23.5 ± 3.69	0.009
Smoking, n (%)	28 (13.3%)	34 (8.3%)	0.087
Alcohol consumption, n (%)	27 (12.9%)	54 (13.2%)	0.913
Comorbidities			
Hypertension	123 (58.6%)	224 (54.6%)	0.35
Diabetes mellitus	79 (37.6%)	112 (27.3%)	0.009
Dyslipidemia	19 (9.0%)	43 (10.5%)	0.572
Heart disease	33 (15.7%)	60 (14.6%)	0.721
Cerebrovascular disease	22 (10.5%)	47 (11.5%)	0.711
History of malignancy	53 (25.2%)	103 (25.1%)	0.975
Chronic lung disease	16 (7.9%)	27 (6.6%)	0.632
History of tuberculosis	12 (5.7%)	20 (4.9%)	0.656
Chronic kidney disease	14 (6.7%)	29 (7.1%)	0.85
Chronic liver disease	8 (3.8%)	9 (2.2%)	0.244
Osteoporosis	8 (3.8%)	29 (7.1%)	0.104
Cognitive impairment	14 (6.7%)	11 (2.7%)	0.017
Parkinson’s disease	8 (3.8%)	6 (1.5%)	0.063
Psychiatric disease	8 (3.8%)	15 (3.7%)	0.925
Benign prostate hyperplasia*	16 (12.8%)	38 (15.8%)	0.491
Number of comorbidities			0.418
0	18 (8.6%)	47 (11.5%)	
1	112 (53.3%)	223 (54.4%)	
≥ 2	80 (38.1%)	140 (34.1%)	
Visual impairment	56 (26.7%)	98 (23.9%)	0.451
Hearing impairment	18 (8.6%)	37 (9.0%)	0.851
Sleep disorder	13 (6.2%)	26 (6.3%)	0.596
Length of hospital stay	19.5 ± 18.8	11.7 ± 9.53	< 0.001
Discharge disposition			< 0.001
Home	137 (65.2%)	343 (83.7%)	
Hospital transfer	63 (30.0%)	46 (11.2%)	
Death	9 (4.3%)	10 (2.4%)	

Data are presented as means (standard deviation) or frequencies (percentage), as appropriate. *The prevalence of benign prostate hyperplasia was calculated in male subjects.

### Comparison of fall risk assessment and laboratory results between fallers and controls

Table 2 shows the comparison of fall risk assessment and laboratory results between the groups. Mean scores of MFS items were higher among fallers than controls (39.3 and 30.4, respectively, *P* < 0.001). Fifty percent of fallers and 32% of controls had a high risk of falling. Among the six items of the MFS, more fallers than controls had a history of falling, ambulatory aid, gait impairment, and altered mental status (*P* = 0.002, *P* = 0.001, *P* = 0.001, and *P* = 0.009). Fallers had higher percentages of leukocytosis, hypoalbuminemia, and hyponatremia compared to controls (22.4% and 13.2%, *P* = 0.003; 54.8% and 46.3%, *P* = 0.047; 30.5% and 22.0%, *P* = 0.020; respectively).

**Table 2:** Fall risk assessment and laboratory results of fallers and matched controls.

	Fallers (n = 210)	Controls (n = 410)	*P*-value
Morse Fall Scale (score)	39.3 ± 22.64	30.4 ± 21.12	< 0.001
Assessment of fall risk			< 0.001
High risk (≥ 45)	105 (50.0%)	131 (32.0%)	
Low risk (< 45)	105 (50.0%)	279 (68.0%)	
Item of Morse Fall Scale			
History of falling within 3 months	30 (14.3%)	27 (6.6%)	0.002
Secondary diagnosis	61 (29.0%)	107 (26.1%)	0.434
IV therapy/heparin lock	146 (69.5%)	256 (62.4%)	0.08
Ambulatory aid			
Crutches, cane, walker	88 (41.9%)	116 (28.3%)	0.001
Furniture	16 (7.6%)	21 (5.1%)	
Gait			
Weak gait	130 (61.9%)	191 (46.6%)	0.001
Impaired gait	4 (1.9%)	13 (3.2%)	
Mental status (forgets limitation)	33 (15.7%)	36 (8.8%)	0.009
Laboratory results			
Leukocytosis (> 10,000/uL)	47 (22.4%)	54 (13.2%)	0.003
Anemia (men < 13 g/dL, women < 12 g/dL)	144 (68.6%)	270 (65.9%)	0.457
Hypoalbuminemia (< 3.8 g/dL)	115 (54.8%)	190 (46.3%)	0.047
Decreased eGFR (< 60 mL/ (min*1.73 m^2^))	47 (22.4%)	95 (23.2%)	0.825
Hyponatremia (< 135 mmol/L)	64 (30.5%)	90 (22.0%)	0.020
Hypokalemia (< 3.6 mmol/L)	34 (16.2%)	49 (12.0%)	0.142
Abnormal liver function test (≥ Upper normal limit*2)	17 (8.1%)	35 (8.5%)	0.851
Uncontrolled diabetes (Hba1c ≥ 8.0%)	19 (9.0%)	24 (5.9%)	0.138

Data are presented as means (standard deviation) or frequencies (percentage), as appropriate.

### Comparison of medications and FRID between fallers and controls

Table 3 shows the comparison of medications and FRID between the groups. Both number of total medications and FRID were higher in fallers than controls (all *P* < 0.001). Among FRID, calcium channel blockers, diuretics (thiazides or spironolactone), antipsychotics, anticonvulsants, benzodiazepine, and antiparkinsonians were more commonly taken by fallers than controls (21.9% and 12.0%, *P* = 0.001; 10% and 4.4%, *P* = 0.006; 17.1% and 11.0%, *P* = 0.031; 26.2% and 12.0%, *P* < 0.001; 10.0% and 4.1%, *P* = 0.004; 8.6% and 4.4%, *P* = 0.004; and 3.9% and 0.7%, *P* = 0.009; respectively). Fallers had higher polypharmacy compared to controls (79.5% and 65.9%, respectively, *P* < 0.001). However, there was no significant difference between the groups based on medication changes on the day before falls (*P* = 0.099).

**Table 3:** Fall risk-increasing drugs prescribed to fallers and matched controls.

	Fallers (n = 210)	Controls (n = 410)	*P* value
Number of medications	8.5 ± 4.55	6.6 ± 4.37	< 0.001
Number of FRID	2.6 ± 2.10	1.8 ± 1.87	< 0.001
FRID			
Digoxin	6 (2.9%)	8 (2.0%)	0.472
Beta blockers	29 (13.8%)	44 (10.7%)	0.26
Calcium channel blockers	46 (21.9%)	49 (12.0%)	0.001
ACE inhibitors or ARBs	46 (21.9%)	70 (17.1%)	0.158
Loop diuretics	33 (15.7%)	48 (11.7%)	0.161
Diuretics (thiazides or spironolactone)	21 (10.0%)	18 (4.4%)	0.006
Alpha blockers	26 (12.4%)	56 (13.7%)	0.657
Anticholinergics	24 (11.4%)	46 (11.2%)	0.938
Antihistamines	18 (8.6%)	25 (6.1%)	0.251
Weak opioids	40 (19.0%)	77 (18.8%)	0.936
Opioids	24 (11.4%)	51 (12.4%)	0.715
Antipsychotics	36 (17.1%)	45 (11.0%)	0.031
Anticonvulsants	55 (26.2%)	49 (12.0%)	< 0.001
Benzodiazepines	21 (10.0%)	17 (4.1%)	0.004
Hypnotics	7 (3.3%)	6 (1.5%)	0.124
Anti-dementia drugs	7 (3.3%)	9 (2.2%)	0.398
Antiparkinsonians	8 (3.9%)	3 (0.7%)	0.009
SSRIs or SNRIs	14 (6.7%)	18 (4.4%)	0.225
TCAs	8 (3.8%)	10 (2.4%)	0.336
Other antidepressants	5 (2.4%)	9 (2.2%)	0.883
Muscle relaxants	15 (7.1%)	19 (4.6%)	0.194
Vasodilating agents	14 (6.7%)	22 (5.4%)	0.512
Polypharmacy (≥ 5 medications)	167 (79.5%)	270 (65.9%)	< 0.001
Medication change on the day before falls			0.099
Inclusion of FRID	76 (36.2%)	115 (28.0%)	
Withdrawal of FRID	15 (7.14%)	36 (8.78%)	

Data are means (standard deviation) or frequencies (percentage), as appropriate.

ACE inhibitors, angiotensin-converting enzyme inhibitors; ARBs, angiotensin II receptor Antagonists; FRID, fall risk-increasing drugs; SSRIs, selective serotonin reuptake inhibitors; SNRIs, serotonin norepinephrine re-uptake inhibitors; TCAs; tricyclic antidepressants.

### Conditional logistic regression analysis of clinical factors and falls

Table 4 presents the association between clinical factors and falls. Using conditional logistic regression analysis, ORs indicated that older adults with diabetes mellitus and high fall risk as assessed by the MFS had 1.76-fold (95% CI 1.12–2.75) and 2.23-fold (95% CI 1.44–3.44) higher risk of falls, respectively. ORs also indicated that among older adults prescribed FRIDs, those taking calcium channel blockers, diuretics (thiazides or spironolactone), anticonvulsants, and benzodiazepines had higher fall risks by 1.71 times (95% CI 1.01–2.88), 2.24 times (95% CI 1.02–4.90), 3.04 times (95% CI 1.73–5.32), and 2.26 times (95% CI 1.05–4.85), respectively.

**Table 4:** Conditional logistic regression analysis of fall-related clinical factors.

	Fall	
	OR	(95% CI)
Body mass index, kg/m^2^	0.97	(0.92–1.03)
Comorbidities		
Diabetes mellitus	1.76	(1.12–2.75)
Cognitive impairment	2.91	(0.97–8.70)
Morse Fall Scale assessment		
High risk (≥ 45)	2.23	(1.44–3.44)
Low risk (< 45)	1	
Laboratory result		
Leukocytosis	1.37	(0.81–2.32)
Hypoalbuminemia	1.37	(0.90–2.09)
Hyponatremia	1.45	(0.92–2.28)
Fall risk-increasing drugs		
Calcium channel blockers	1.71	(1.01–2.88)
Diuretics (thiazides or spironolactone)	2.24	(1.02–4.90)
Antipsychotics	1.25	(0.68–2.28)
Anticonvulsants	3.04	(1.73–5.32)
Benzodiazepines	2.26	(1.05–4.85)
Antiparkinsonians	2.80	(0.68–11.49)

Odds ratio (OR) and 95% confidence interval (CI) by conditional logistic regression analysis.

### ROC curves of the clinical factors predicting falls

**Figure 1:**
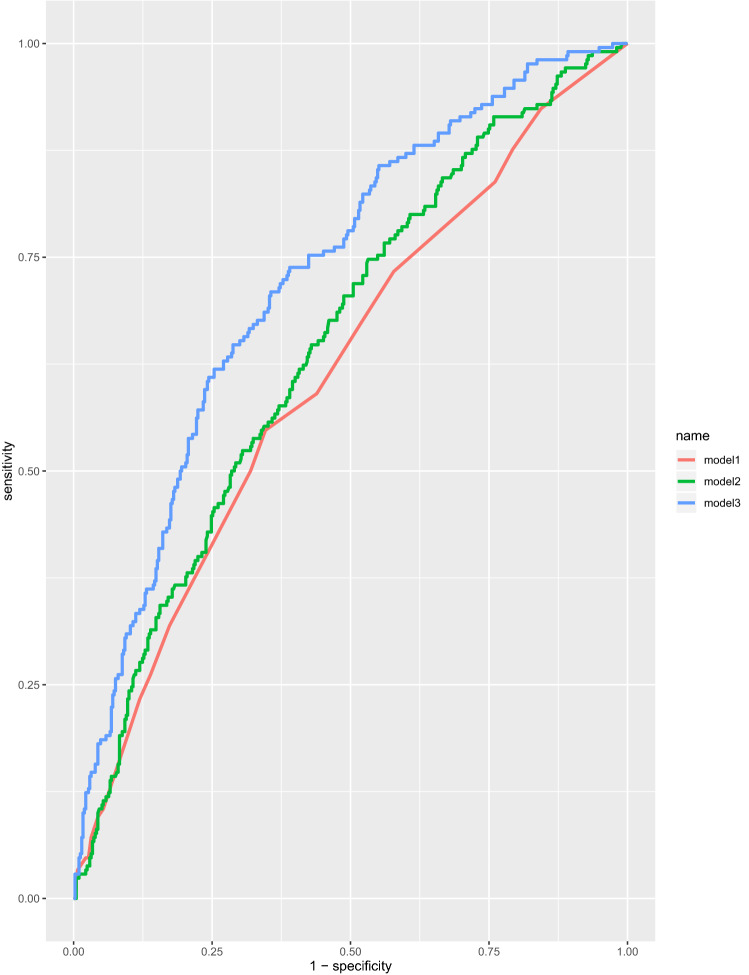
Comparisons of receiver operating characteristic curves. Model 1, (M1) Morse Fall Scale (MFS); Model 2, (M2) MFS, age, sex, ward, and polypharmacy; Model 3, (M3) MFS, age, sex, ward, polypharmacy, diabetes mellitus, cognitive impairment, leukocytosis, hypoalbuminemia, hyponatremia, calcium channel blockers, diuretics (thiazides or spironolactone), antipsychotics, anticonvulsants, benzodiazepines, and antiparkinsonians.

Figure 1 shows the comparisons of three ROC curves of the clinical factors predicting falls. M1 was MFS only. Age, sex, ward, and polypharmacy were added in M2, and diabetes mellitus, cognitive impairment, leukocytosis, hypoalbuminemia, hyponatremia, calcium channel blocker, diuretics (thiazides or spironolactone), antipsychotics, anticonvulsants, benzodiazepines, and antiparkinsonians were added in M3. The AUC (95% CI) of each model was 0.615 (0.568–0.662), 0.646 (0.601–0.692), and 0.725 (0.683–0.766), respectively. There were significant improvements in discrimination between fall cases and controls when clinical factors were combined with MFS (M1 vs. M2, *P* = 0.042; M2 vs. M3, *P* < 0.001, respectively).

## Discussion

In this matched case–control study, we demonstrated that several clinical factors also identified in other recent studies were associated with inpatient falls. After adjusting for the MFS, diabetes mellitus and FRID, including calcium channel blockers, diuretics (thiazides or spironolactone), anticonvulsants, and benzodiazepines, were significantly associated with higher fall risk. Additionally, we identified significant improvements in the accuracy of fall risk prediction when clinical factors were combined with MFS.

In our study, diabetes mellitus was associated with the risk of falling. A previous study based on data from the longitudinal Health, Aging, and Body Composition Study reported that older adults with diabetes mellitus had higher risks of injurious falls^19^. Risk factors for falls including peripheral neuropathy, visual impairment, and decreased physical performance are more common in patients diagnosed as diabetic^20^. Our results concerning older patients showed that the prevalence of cognitive impairment was also higher in fallers than controls, although there was no significant association identified by conditional logistic regression analysis. Older adults experiencing dementia were also at a higher risk of falling^21^. This makes sense in view of a systematic review and meta-analysis that reported that cognitive impairment was associated with falls, injurious falls, and distal radius fractures^22^. Furthermore, in older adults with dementia, the decline of motor and executive function, neuropsychiatric symptoms, and related medication use were associated with fall risk^23,24^.

Among FRID, in our study, calcium channel blockers, diuretics excluding loop diuretics, anticonvulsants, and benzodiazepines were significantly associated with fall risk. The association of calcium channel blockers and diuretics with fall risk is controversial. A recent systematic review and meta-analysis including five studies reported that taking calcium channel blockers was not associated with fall risk (OR 1.00, 95% CI 0.80–1.24)^25^. However, among the five studies, one that was conducted with patients in acute care hospitals reported that calcium channel blockers were significantly associated with fall risk (OR 2.45, 95% CI 1.20–5.01)^26^, which was consistent with our findings. Older adults commonly use diuretics as antihypertensives and for treating and preventing heart failure as well as ascites in liver cirrhosis. However, they can cause orthostatic hypotension, dizziness, and muscle weakness related to hyponatremia, which lead to increased risks of falls, and, in our study, diuretics excluding loop diuretics were associated with increased fall risk. In fact, another population-based case–control study reported that thiazides increased fall risk (OR 1.25, 95% CI 1.15–1.36)^27^, in line with our findings. However, recent systematic reviews and meta-analyses have also reported that loop diuretics are associated with fall risk, whereas thiazides are not^28^. Therefore, there is a need for further studies to clarify the actual fall risk associated with this drug in hospitalized older adults.

We also noted that many of the older adults in our study used anticonvulsants for treating epilepsy, mood disorders, and neuropathic pain^29^. Adverse reactions to antiepileptic drugs including dizziness, blurred vision, and sedation are likely to increase the risk of falls, as demonstrated in a recent systematic review and meta-analysis that reported an association between antiepileptic use and increased risk of falling in older adults^30^. We also looked at the use of benzodiazepines, and the results indicate that they are associated with higher fall risk, which is in line with a previous systematic review^31^. Benzodiazepines can cause sedation and impaired balance and gait, all of which lead to increases in the risk of falls among older adults^32^.

Our results also indicate that the prevalence of leukocytosis, hypoalbuminemia, and hyponatremia was higher in fallers than controls. However, there was no significant association between laboratory results and fall risk indicated by conditional logistic regression analysis. Hyponatremia is an electrolyte imbalance common in older adults, which can lead to muscle weakness or cramps, lethargy, and confusion. It can be caused by the use of diuretics and anticonvulsants, and comorbidities include heart failure, cirrhosis, and chronic kidney disease. Previous studies have suggested that hyponatremia is a potential risk factor for falls^13,32^. Hypoalbuminemia, a marker of poor nutritional status, is a possible risk factor for falling. Further, a recent study reported that in hospitalized patients with hematologic diseases, hypoalbuminemia was associated with increased risks of inpatient falls^33^. In French community-dwelling older adults, poor nutritional status, as assessed by the Mini Nutritional Assessment, was associated with falls and fractures^34^. Additionally, leukocytosis, one of the signs of infection, might be associated with fall risk, as a previous study of German older hospitalized patients found that the presence of leukocytosis on admission was associated with falls^11^.

This study had several strengths. First, we were able to identify various fall-related clinical factors before fall events using the CDW. Use of the CDW allowed relatively short data collection times and ensured high data quality^35^. In addition, we analyzed risk-discriminative performances between the MFS alone and MFS plus clinical factors. Second, we focused on hospitalized older adults, a vulnerable population regarding both falls and possible adverse reactions to FRID. However, our study also had some limitations. First, the setting was a single Korean tertiary hospital, which might not be representative of the general population. Second, it is difficult to completely distinguish the effects of comorbidities, related medications, and laboratory results. For example, it is hard to sort out the effects of comorbidities from those of the related medications (cognitive impairment and psychotropic agents) or the relationship of laboratory results and adverse reactions to medications (hyponatremia and diuretics or anticonvulsants). Finally, although we considered comprehensive factors, the AUC of the final model was 0.726, which is not very high, indicating that there is a need for further research to identify other possible fall-related factors. Recently, machine learning algorithms using electronic health records have been evaluated for predicting falls among older adults making emergency department visits^36^. In the near future, developing comprehensive machine learning models to predict inpatient falls based on electronic health records is needed.

In conclusion, we identified that several clinical factors were associated with higher risks of falls among older adults hospitalized for acute care. Patients with a history of diabetes mellitus and the use of calcium channel blockers, diuretics, anticonvulsants, and benzodiazepines were associated with higher fall risks. Adding these clinical factors to the MFS led to significant improvements in fall prediction. Based on the results, we believe that developing novel fall risk assessment tools reflecting the various clinical factors identified is necessary.

## Data Availability

The datasets generated and analyzed during the current study are available from the corresponding author on reasonable request.
